# Low expression of OXCT1 promote colorectal cancer liver metastasis by upregulating CDK8 and β-catenin via H3 acetylation

**DOI:** 10.1016/j.gendis.2025.101625

**Published:** 2025-04-09

**Authors:** Chenhao Li, Deao Gong, Xiaoqun Shan, Kang Wu, Jiayao Yang, Rong Zhang, Ye Huang, Kai Wang, Ni Tang, Yuxi Zhu

**Affiliations:** a Department of Oncology, Laboratory of Immunity, Inflammation & Cancer, The First Affiliated Hospital of Chongqing Medical University, Chongqing 400016, China; b Key Laboratory of Molecular Biology for Infectious Diseases (Ministry of Education), Institute for Viral Hepatitis, Department of Infectious Diseases, The Second Affiliated Hospital, Chongqing Medical University, Chongqing 400010, China; c Chongqing Medical University, Chongqing 400016, China

**Keywords:** Bioinformatics analysis, Colorectal cancer, Liver metastasis, OXCT1, Wnt signaling pathway

## Abstract

Colorectal cancer (CRC) poses a significant global health challenge, with liver metastasis being a major contributor to its high mortality rate. The liver, due to its strategic anatomical location, distinctive tissue architecture, and unique metabolic properties, is the primary site for CRC to metastasize. The objective of this study was to identify hub genes involved in colorectal liver metastasis (CRLM) using a combination of bioinformatics analysis and experimental methods, and to decipher their molecular mechanisms that regulate this metastatic process. By mining data from the TCGA and GEO databases, we identified that low expression of 3-ketoacid CoA transferase 1 (OXCT1) might related to the development of CRLM. Both *in vitro* and *in vivo* experiments have indicated that the downregulation of OXCT1 significantly enhanced tumor migration and metastasis, suggesting a potential tumor-suppressive role for OXCT1 in the progression of CRLM. Further bioinformatics analysis, dual-luciferase reporter assays, and Western blot identified Yin Yang 1 (YY1) as a transcription factor regulating OXCT1 in CRC. RNA sequencing suggested that OXCT1 suppressed the Wnt signaling pathway by downregulating CDK8 expression, and diminishing its interaction with β-catenin. Additionally, OXCT1 governed CDK8 expression via histone H3 acetylation. Finally, OXCT1 expression was significantly reduced in CRLM sites, which correlated with unfavorable outcomes. Our research suggested the OXCT1/Wnt signaling axis pathway as a critical regulator of CRLM. And these findings offered valuable insights, and potential therapeutic targets for CRLM.

## Introduction

Colorectal cancer (CRC) has become one of the most prevalent and deadly malignancies worldwide.^1^^,^^2^ It is characterized by a propensity for metastasis to the liver, a condition known as colorectal cancer liver metastasis (CRLM), which affects approximately 20%–30% of patients throughout the disease trajectory.^3^ Previously, the mechanisms underlying CRLM have been mainly attributed to anatomical predispositions and immune suppression.^4^ However, recent evidence suggests that additional, yet unidentified factors may contribute to the development of CRLM.

The prognosis for patients with CRLM is grim, with a five-year survival rate ranging from 12% to 30%. Most patients with CRC liver metastases who do not receive treatment typically have a life expectancy ranging from 6 to 20 months.^2^ Therefore, in-depth exploration of the new mechanisms underlying CRLM, as well as the formation of metastatic foci is critical for identifying therapeutic targets, intervening in early liver metastasis progression, and improving prognosis.

Locally advanced CRC frequently invades surrounding tissues, a process that is heavily influenced by tumor cell motility—a key determinant of metastatic potential.^5^ Recently, several key molecules, including PCDH17^6^ and lncRNA XIST,^7^ have been identified as being involved in this process. Moreover, aberrant activation of the Wnt signaling pathway is frequently observed in solid tumors.^8^ The loss of normal cell polarity and adhesion caused by Wnt signaling activation is a fundamental step in tumor progression and metastasis.^9^ Therefore, understanding the mechanisms of Wnt signaling activation and targeting tumor cell motility through Wnt signaling inhibition may serve as an effective strategy for treating high-risk metastatic cancers.^10^^,^^11^

In this study, we employed bioinformatics analysis to identify 3-oxoacid CoA-transferase 1 (OXCT1) as a pivotal gene that may regulate CRLM. We also validated OXCT1's role in the biological functions of colorectal tumor cells through both in vivo and in vitro experiments. Moreover, we performed transcriptome sequencing analysis, and revealed that OXCT1 significantly influences the Wnt signaling pathway, particularly affecting the CDK8 gene. Finally, we discovered that OXCT1 epigenetically regulates CDK8 transcription. This study highlights the essential regulatory role of OXCT1 in CRLM, elucidates its specific molecular mechanisms, and offers a novel potential target for preventing CRLM.

## Materials and methods

### Data acquisition and processing

Transcriptome data related to colorectal cancer metastasis and corresponding clinical information were downloaded from the Gene Expression Omnibus (GEO) and The Cancer Genome Atlas (TCGA) databases (Table S1). Differential gene expression analysis was performed using the limma^12^ package in the R programming environment, comparing primary tumors and liver metastasis groups in the GSE41258, GSE68468, and GSE35834 datasets. In the GSE41258 dataset, genes were considered significantly differentially expressed with a threshold of p.adj < 0.05 and |logFC| ≥ 1. For the GSE68468 and GSE35834 datasets, given the lower number of differentially expressed genes, thresholds were adjusted to p.adj < 0.05 and |logFC| ≥ 0.5. Subsequently, volcano plots were generated using the ggplot2 package.

Univariate COX regression analysis was conducted on the differentially expressed genes from the GSE41258 dataset using the survival package. Genes with *p* < 0.05 were subjected to the subsequent lasso regression. Genes with a hazard ratio (HR) greater than 1 were classified as risk factors for colorectal cancer prognosis, whereas those with HR less than 1 were deemed protective factors. The glmnet and survival packages were then used to further filter the candidate genes, and genes with *p* < 0.05 were incorporated into a risk model and identified as key genes in colorectal cancer metastasis.

### Clinical sample collection and immuno-histochemistry experiments

Paraffin-embedded tissue sections of cancerous and adjacent non-cancerous tissues were collected from 20 colon cancer patients, obtained from the Department of Pathology at Chongqing Medical University. The sections were incubated overnight at 4 °C with a primary antibody against OXCT1. Subsequently, the sections were incubated with a secondary antibody, anti-rabbit IgG (ZSGB-BIO, Beijing, China) and stained using 3,3′-diaminobenzidine (DAB). The stained sections were scanned using a Pannoramic Scan 250 Flash scanner, and the images were acquired using Pannoramic Viewer 1.15.2 software (3DHistech, Budapest, Hungary).

### Cell cultures and reagents

Human cell lines HCT116 and RKO were obtained from the Cell Bank at the Chinese Academy of Sciences (Shanghai, China). Mycoplasma contamination was ruled out using the MycoAlert PLUS kit (Lonza, based in Basel, Switzerland). Cells were cultured in Dulbecco's Modified Eagle Medium (DMEM; Gibco, Grand Island, NY, USA). This medium was supplemented with 10% fetal bovine serum (FBS; Corning, NY, USA), 100 mg/mL streptomycin, and 100 IU/mL penicillin (MCE, NJ, USA). The cultures were maintained at 37 °C in an humidified atmosphere of 5% CO_2_.

### Adenovirus production and stable cell lines construction

The amplified OXCT1, OXCT1-S226N, YY1 fragments were cloned into the shuttle vector pAdTrack-TO4, provided by Dr. He Tong-Chuan (University of Chicago, USA). Recombinant adenovirus pAd-OXCT1 was generated using the AdEasy system, with AdGFP was used as a negative control as previously described.^13^

The CRISPR-Cas9 system, provided by Professor Ding Xue (Tsinghua University, Beijing, China), was used to construct OXCT1 knockout (KO) cells as previously described.^14^ The KO efficiency was verified by Western blot analysis.

### Quantitative real-time PCR

Total RNAs were extracted from colorectal cancer cells using TRIzol reagent (Invitrogen, Rockville, MD, USA) and reverse transcribed into cDNA with the PrimeScript™ RT reagent kit (RR047A, TaKaRa, Tokyo, Japan). Quantitative real-time PCR was conducted on a Bio-Rad CFX96 system (Bio-Rad, Hercules, CA, USA), with β-actin serving as the normalization control to calculate mRNA levels via the 2-ΔΔCt method.

### Western blot

Cells were lysed with a cold buffer containing 1 mM phenylmethylsulfonyl fluoride (PMSF) from Beyotime Biotechnology (Shanghai, China). The resulting cell suspension was kept on ice and vortexed for 20 min. Sonication was then performed. The cell suspension was then centrifuged at 14,000 rpm for 20 min at 4 °C. The protein concentration in the supernatant was measured using the BCA Protein Assay Kit (Beyotime Biotechnology). Protein extracts were resolved by 10% or 12% SDS-PAGE and subsequently transferred onto PVDF membranes (Millipore, Bedford, MA). Membranes were incubated in a blocking solution of 5% non-fat milk in TBST for 1.5 h, followed by overnight incubation with the specified primary antibodies at 4 °C. After washing, membranes were incubated with secondary antibodies (Abcam, Cambridge, UK), and immunoreactive bands were visualized using the Enhanced Chemiluminescence (ECL) system (Bio-Rad).

### Proliferation assay

Colon cells were plated in 96-well plates at a density of 800 cells per well. Each experimental condition was replicated three times and incubated for five days. The growth rate of the cells was monitored daily by using the IncuCyte ZOOM software (ESCO, USA).

### Transwell migration assay

Cell migration was assessed using 24-well Transwell plates (Corning, USA) with a serum concentration gradient established between chambers. The upper compartments were inoculated with 5 × 10^4^ cells suspended in 200 μL of serum-deprived medium, while the lower compartments contained 800 μL of complete medium supplemented with 10% fetal bovine serum. After incubation, membrane-traversing cells were fixed and subjected to crystal violet staining. Cellular quantification was performed by counting stained cells in three randomly selected microscopic fields per insert under 200 × magnification using a ZEISS Axio Imager A2 microscope (Germany).

### Wound healing assay

Cells were cultivated in 96-well plates, and a scratch was made in the cell monolayer using a WoundMaker (ESSEN). ESCO IncuCyte ZOOM software documented the area of the wound, and the extent of scratch closure was calculated.

### Cecum injection

In the cecal injection model, 6-week-old nude mice were anesthetized with 2.5% sodium pentobarbital (40 mg/kg) and then subjected to a surgical incision. HCT116 cells (2 × 10^6^) from the AdGFP, AdOXCT1, sgControl, and OXCT1-KO groups (5 mice per group) were injected into the cecal wall, followed by closure of the mice with sterile sutures. Six weeks later, the size and number of liver metastases were observed, the liver metastasis was assessed by weighing and the tumor area was analyzed using imageJ.

### Chromatin immunoprecipitation (ChIP) assay

Chromatin immunoprecipitation was performed using chemically crosslinked HCT116 cells (8 × 10^6^ cells fixed with 1% paraformaldehyde at 37 °C, 8 min). Chromatin fragmentation was achieved through ultrasonic processing (30% amplitude, 10 cycles of 15-sec pulses interspersed with 15-sec intervals). Immunoenrichment was conducted by incubating clarified lysates with specific anti-YY1 (SC-7341, Santa Cruz) or isotype-matched control antibodies in ChIP dilution buffer (16 h, 4 °C). Chromatin-immunocomplexes were captured with Protein A/G magnetic beads (Millipore, 16-663), followed by sequential washes with low-salt, high-salt, and hiTE buffers. The DNA fragments bound to YY1 in the ChIP samples were then analyzed using real-time PCR. The procedures for ChIP and ChIP-qPCR were performed in accordance with previously established protocols.^14^

### Dual-luciferase reporter assay

The OXCT1 promoter sequence (fragment +878 to +2100) was cloned into the pGL3-basic plasmid to investigate the interaction between YY1 and the OXCT1 promoter. HCT116 and RKO cells were transfected with AdTrack-TO4 (used as a negative control) or YY1-TO4 for 24 h in 12-well plates. Subsequently, cells were co-transfected with 0.5 μg of pGL3-OXCT1 and 25 ng of pRL-TK-Renilla (used as a transfection control) for 48 h. Luciferase activity was then measured using the Dual-Luciferase Reporter Assay System (Promega, Madison, WI, USA).

To assess the transcriptional activity of β-catenin downstream target genes, we utilized the TOPflash and FOPflash luciferase reporter assay (Beyotime, China). HCT116 and RKO cells were infected with AdGFP (negative control) or AdOXCT1 for 24 h, or seeded with sgControl, OXCT1-KO1, or OXCT1-KO2. The cells were then co-transfected with 0.5 μg of the TOPflash or FOPflash plasmid and 25 ng of the pRL-TK-Renilla plasmid for 48 h. Luciferase activity was subsequently measured using the Dual-Luciferase Reporter Assay System.

### RNA-seq

HCT116 cells (1 × 10^7^) were treated with either Ad-GFP or Ad-OXCT1 for 36 h to perform RNA sequencing (RNA-Seq). Following the manufacturer's instructions, total RNA was extracted using TRIzol reagent (Invitrogen), and the RNA-seq experiment was then conducted by Applied Protein Technology (Shanghai, China).

### Co-immunoprecipitation (Co-IP)

Co-immunoprecipitation (co-IP) assays were performed by incubating 500 μg total protein lysates with specific primary antibodies (1 μg) in IP lysis buffer (4 °C, 12h, rotating platform). Antigen-antibody complexes were precipitated using Protein A/G Plus Agarose (MCE, HY-K0202), followed by three washes with ice-cold PBS containing 0.1% Tween-20. Bound proteins were denatured in 2 × loading buffer (95 °C, 5 min), resolved on 10% SDS-PAGE gels, and immunoblotted with species-matched secondary antibodies.

### Human acetyl-coenzyme A ELISA kit

To quantify acetyl-CoA levels, cells or tissues were rinsed with chilled PBS, collected in RIPA buffer, and subjected to sonication. Proteins from the cellular or tissue samples were then precipitated using PCA. Following centrifugation, the supernatant was collected and adjusted to a neutral pH (6-8) by adding potassium bicarbonate. Any resulting precipitates were eliminated through additional centrifugation. The final supernatant was analyzed in triplicate for acetyl-CoA content using the COIBO CB12747-HU assay kit, adhering to the manufacturer's guidelines.

### Statistical analysis

Data analysis was performed using R software (version 4.1.1). Differences between groups were tested using t-tests, one-way ANOVA, and *χ*^2^ tests. For differential gene expression analysis, *p*-values were adjusted using the Benjamini-Hochberg (BH) procedure, with an adjusted *p*-value threshold of less than 0.05 considered statistically significant. ∗*p* < 0.05, ∗∗*p* < 0.01, ∗∗∗*p* < 0.001.

## Results

### Low expression of OXCT1 was associated with liver metastasis in colorectal cancer

**Figure S1:**
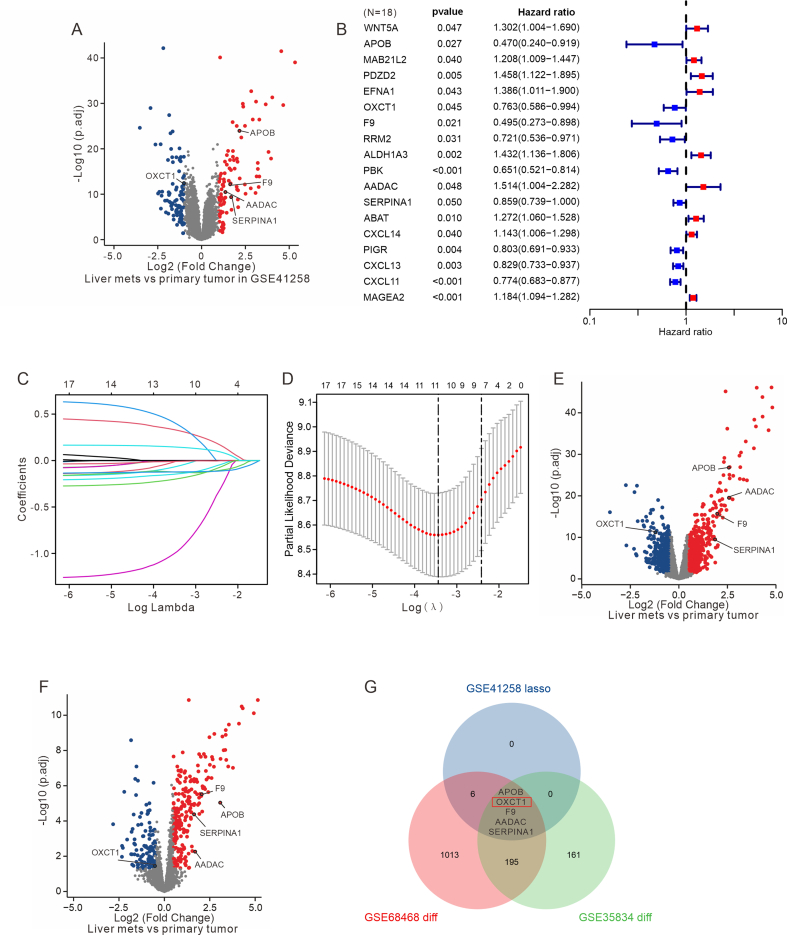
Low expression of OXCT1 was associated with liver metastasis in colorectal cancer. (A) In the GSE41258 cohort, genes with significant differences between the metastatic group and the primary tumor group. (B) Genes that have a significant impact on prognosis obtained from univariate COX analysis of differential genes. (C-D) Lasso regression analysis identified 11 prognostically significant differential genes. (E-F) Volcano plots of expression differential analysis for GSE68468 (E) and GSE35834 (F). (G) Intersection of differentially expressed genes from GSE68468 and GSE35834 with prognostic genes screened by Lasso regression analysis.

To identify the key genes regulating liver metastasis in colorectal cancer, we employed bioinformatics methods for screening and analysis. In the GSE41258 dataset, we conducted a differential gene expression analysis between primary tumor samples and liver metastasis samples, visualizing the results with a volcano plot (Fig. S1A). We identified 190 genes with significantly altered expression levels. Subsequent Univariate Cox regression analysis revealed that 18 of these genes were significantly associated with colorectal cancer liver metastasis and the prognosis of patients (Fig. S1B).

To refine the selection of prognostic genes, we applied Lasso regression analysis by utilizing the model with the minimum deviance value. Lasso regression is particularly suited for handling high-throughput datasets in cancer biology. When the number of genes far exceeds the number of samples, Lasso effectively selects relevant feature genes, thereby enhancing the predictive accuracy for the target variable.^15^ This analysis identified 11 the variables with non-zero coefficients, as depicted in a coefficient trajectory plot during logarithmic changes in the Lasso penalty parameter (λ) (Fig. S1C-D). These 11 key genes were significantly related to the prognosis of patients.

**Figure 1:**
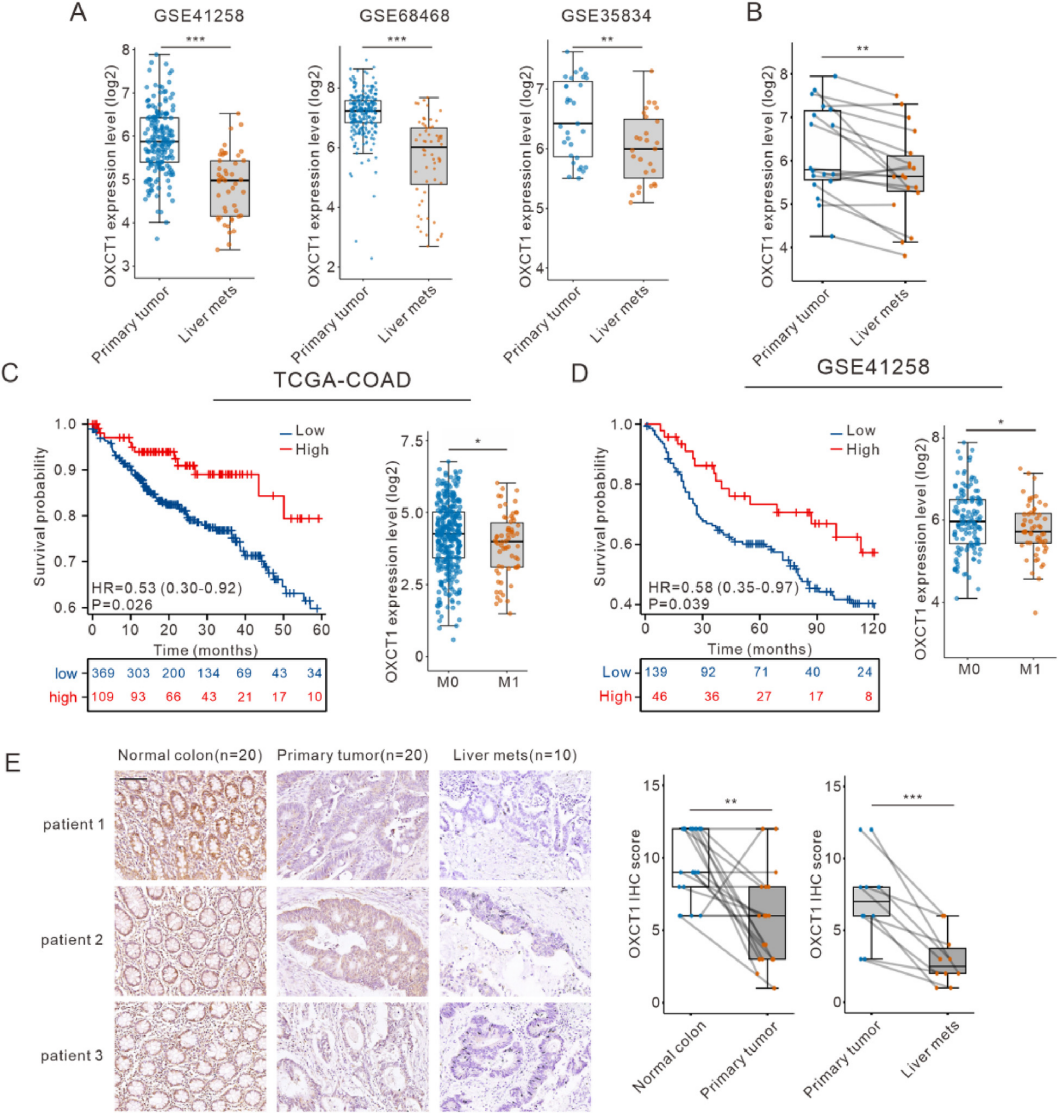
Low expression of OXCT1 was associated with liver metastasis in colorectal cancer. (A) It was shown low expression of OXCT1 in liver metastasis in datasets GSE41258, GSE68468, GSE35834. Student's t - test was used to calculate *p* value. (B) The expression of OXCT1 was low in paired data from GSE41258, GSE68468, GSE35834. Paired two - tailed Student's t - test was used to calculate *p* value. (C–D) Kaplan–Meier analysis of survival curve of patients with CRC in TCGA and GSE41258 with low and high OXCT1 levels. *p* value was calculated based on log-rank test. (E) Representative immunohistochemistry images and evaluation of OXCT1 expression measured on a tissue that contained 20 paired normal colon, primary tumors, and liver metastases. *p* value was calculated based on paired Student's t test. Scale bar: 100 μm (∗*p* < 0.05, ∗∗*p* < 0.01, ∗∗∗*p* < 0.001).

Additionally, we performed differential expression analysis on the GSE68468 and GSE35834 datasets (Fig. S1E-F), and intersected the significantly altered with those previously prognosis-related genes (Fig. S1G). A Venn diagram visualization highlights the intersection, featuring the OXCT1 gene, which is downregulated in colorectal cancer within the datasets and exhibits a hazard ratio (HR) less than 1, suggesting a potential protective role. We then analyzed OXCT1 expression in primary tumors and liver metastases across the GSE41258, GSE68468, and GSE36834 datasets to further validate differential expression using paired patient data (Fig. 1A–B). The results showed a significant downregulation of OXCT1 in liver metastases compared to primary tumors. OXCT1, a key rate-limiting enzyme in ketone body metabolism, is generally associated with poor prognosis when highly expressed in certain cancers, such as liver cancer^16^ and pancreatic cancer.^17^ Interestingly, our findings suggest that it may be downregulated in colorectal cancer, indicating a potential discrepancy. To address this discrepancy, we sought to confirm the potential role of OXCT1 in the liver metastasis of colorectal cancer.

Subsequently, we extracted survival data from the TCGA-COAD (The Cancer Genome Atlas Colon Adenocarcinoma) and GSE41258 datasets for primary tumors to draw Kaplan–Meier survival curves. We also assessed the relationship between the expression level of OXCT1 and colorectal cancer prognosis. The results indicated that patients with low expression of OXCT1 had a poorer prognosis. Further analysis revealed that OXCT1 expression was significantly downregulated in samples from the M1 stage compared with those from the M0 stage (Fig. 1C–D). The M0 stage refers to the absence of distant metastasis, while the M1 stage indicates the presence of distant metastasis.

Finally, we collected tissue sections from 20 patients, including 10 cases with liver metastasis, and 10 cases without liver metastasis. Immunohistochemistry (IHC) analysis confirmed that OXCT1 expression was significantly reduced in liver metastases compared to primary tumors and normal tissues (Fig. 1E). These findings suggested that low OXCT1 expression might be associated with liver metastasis in colorectal cancer.

### OXCT1 promotes colon cancer cells migration in vivo and vitro

**Figure 2:**
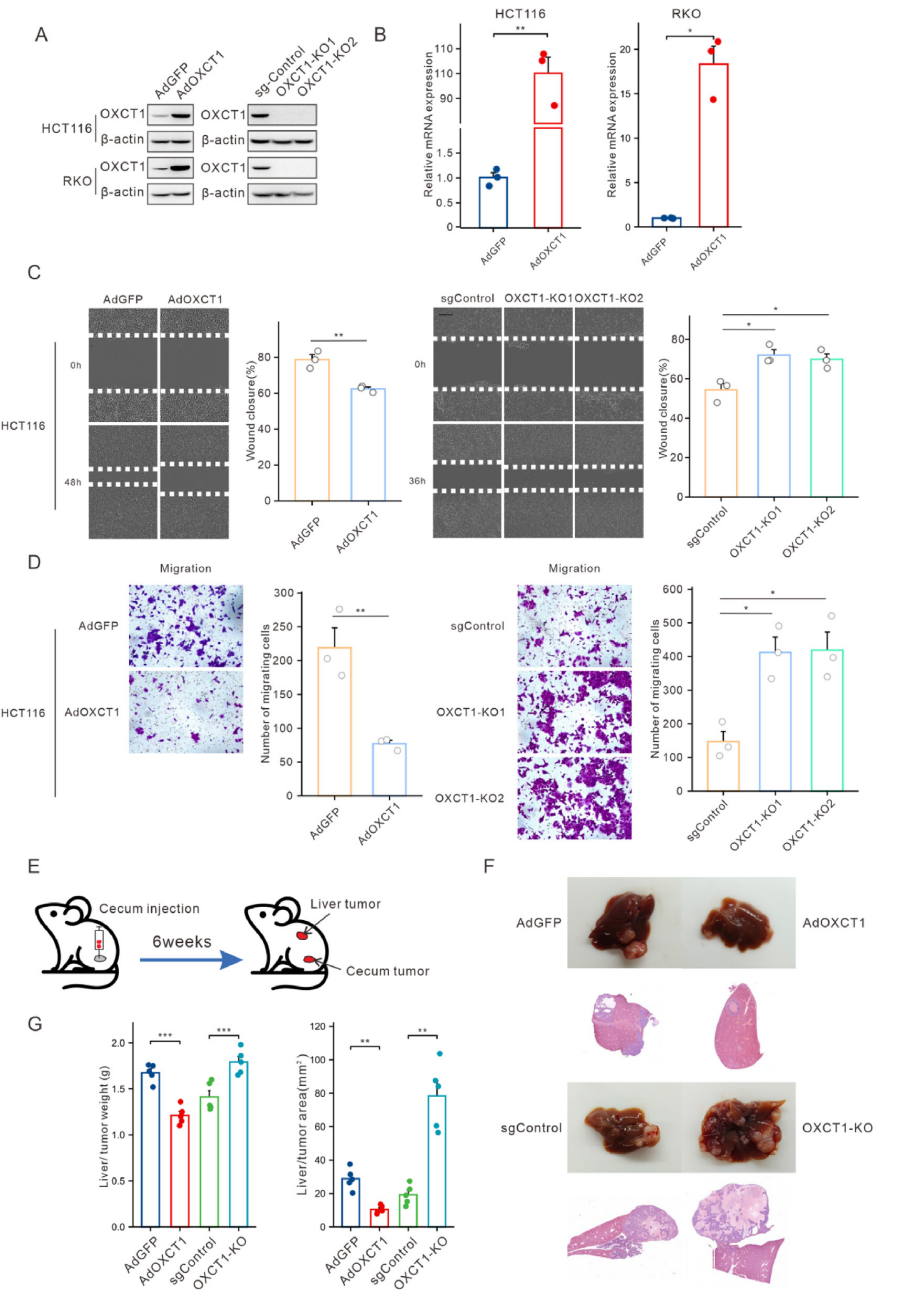
OXCT1 promoted colon cancer cells migration in vivo and in vitro. (A–B) OXCT1 overexpression and knockout system was constructed, validated through Western blot (A) and qRT-PCR (B). (C) Scratch assay was used to assess the impact of OXCT1 on the migratory capacity of HCT116 cells. (D) Transwell experiment was conducted to assess the effect of OXCT1 on the migratory capacity of HCT116 cells. (E) Schematic of the CRC orthotopic mouse model. HCT116 cells were initially injected into the mouse cecum wall, and metastasized to the liver. (F–G) Representative images of liver metastases in the HCT116 mouse cecal injection model of colorectal cancer, along with H&E staining images and corresponding quantitative analysis. The p - value was calculated using a T - test (∗*p* < 0.05, ∗∗*p* < 0.01, ∗∗∗*p* < 0.001).

Next, we constructed the recombinant adenovirus to overexpress OXCT1 in the HCT116 and RKO cell lines. The overexpression was confirmed at both the protein and mRNA levels using Western blot and qRT-PCR analyses (Fig. 2A–B). In parallel, we established OXCT1 stable knockout cell lines in HCT116 and RKO cells by CRISPR-Cas9 technology. The efficiency of the knockout was validated using Western blot analysis (Fig. 2A).

**Figure S2:**
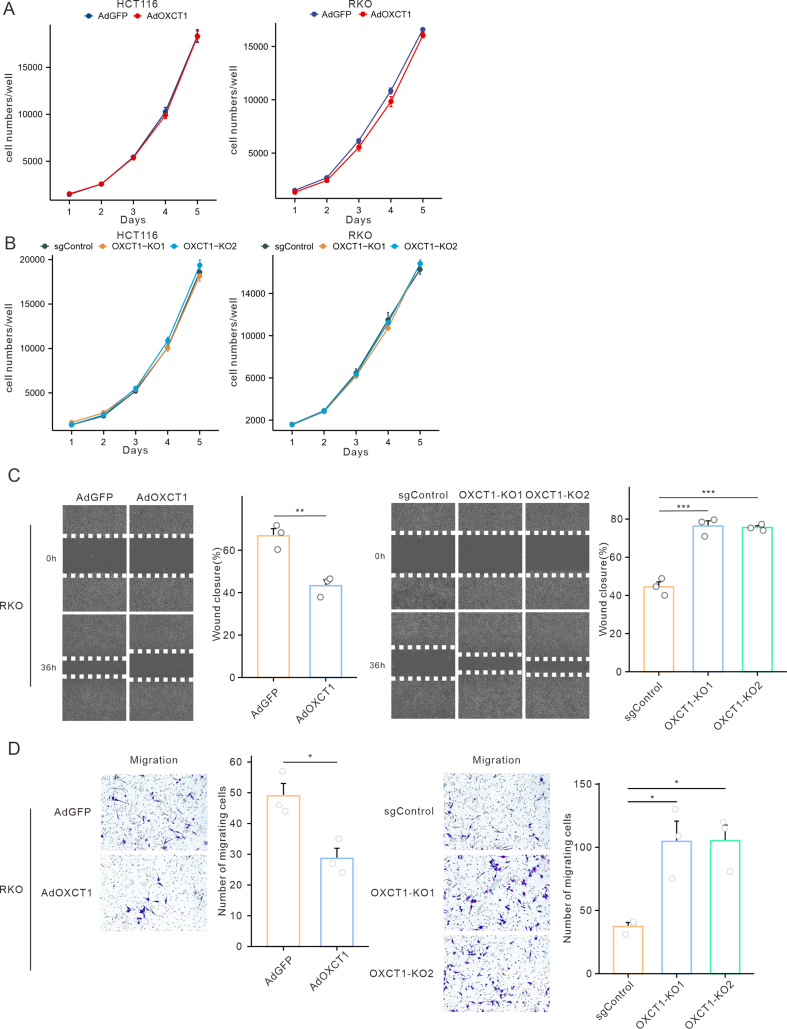
OXCT1 promoted colon cancer cells migration in vivo and in vitro. (A-B) The cell proliferation curve indicated that overexpression (A) and knockout (B) of OXCT1 do not affect the proliferation in HCT116 and RKO. (C) Scratch assay was used to assess the impact of OXCT1 on the migratory capacity of RKO. (D) Transwell experiment was conducted to assess the effect of OXCT1 on the migratory capacity of RKO (∗*p* < 0.05, ∗∗*p* < 0.01, ∗∗∗*p* < 0.001).

To further investigate the impact of OXCT1 on cell biological function, we conducted cell proliferation assays. Cell growth curves indicated no significant difference in cell number between the AdOXCT1-and AdGFP-transfected groups (Fig. S2A). Similarly, proliferation assays following OXCT1 knockout showed no significant change in proliferative capacity among the OXCT1-KO1, OXCT1-KO2, and the sgControl groups (Fig. S2B). These findings indicated that OXCT1 expression levels do not substantially affect the proliferation of HCT116 and RKO cells in vitro.

To further explore the role of OXCT1 on cell migration, wound healing assays were performed in HCT116 and RKO cells. The results revealed that OXCT1 overexpression significantly impaired the migration capacity of colon cancer cells (Fig. 2C, Fig. S2C), while OXCT1 knockout significantly promoted cell migration. Moreover, this finding was corroborated by transwell assays, which showed that OXCT1 overexpression reduced the migration of both HCT116 and RKO cells, whereas OXCT1 knockout enhanced their migration (Fig. 2D, Fig. S2D).

To investigate the effect of OXCT1 on colon cancer metastasis in vivo, we employed a cecum injection model with orthotopic cecum implantation^18^ using HCT116 cells, followed by assessment of liver metastasis (Fig. 2E). Six weeks following the procedure, liver samples were collected from the mice for analysis. The results showed that OXCT1 overexpression significantly reduced the number of liver metastases, while OXCT1 knockout significantly increased them (Fig. 2F–G). These findings suggest that OXCT1 plays an inhibitory role in the metastasis of colon cancer in vivo.

### The transcription regulation of OXCT1 by Yin and Yang1 (YY1)

**Figure 3:**
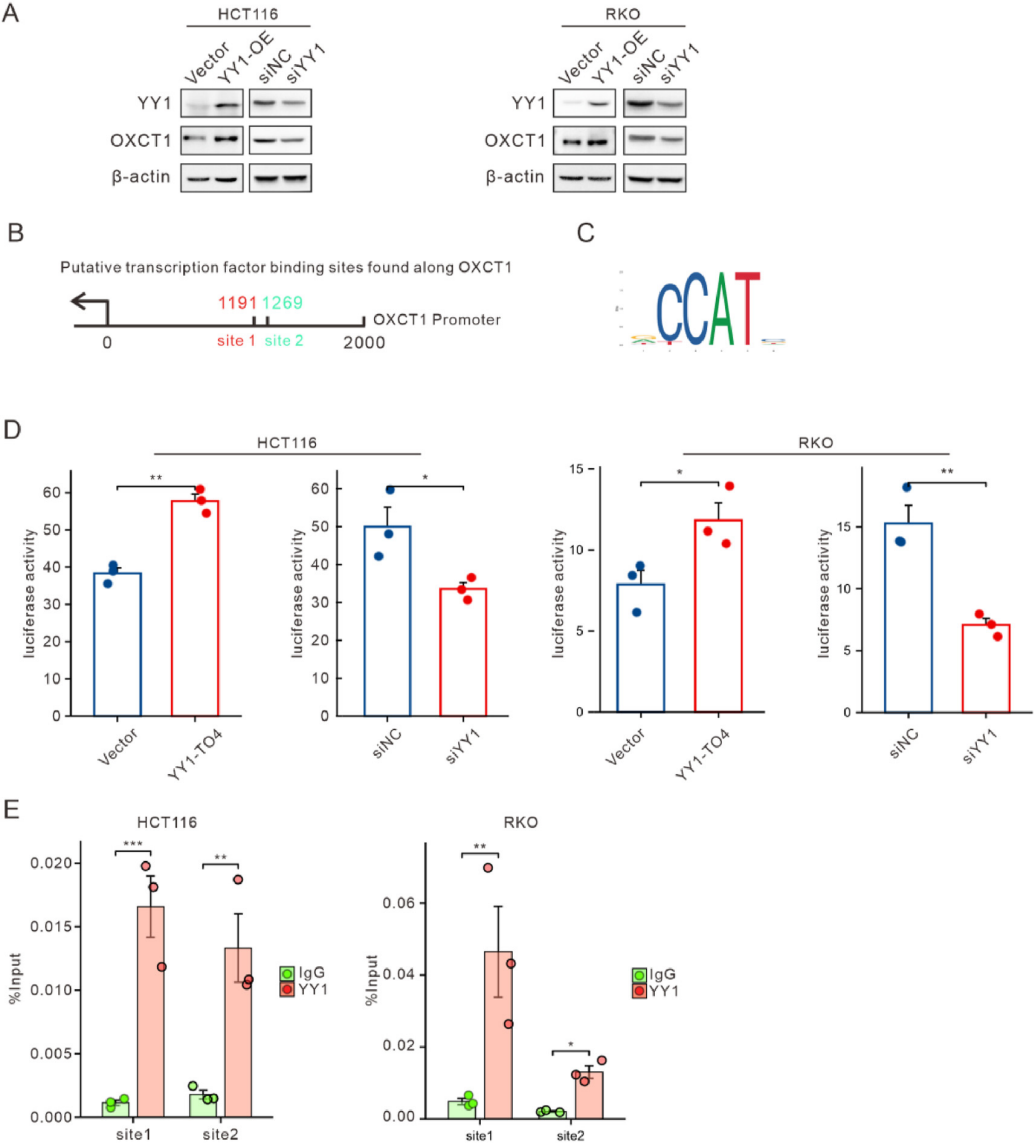
The transcription regulation of OXCT1 by YY1. (A) The protein levels of OXCT1 were regulated by YY1 in HCT116 and RKO cells. (B) Schematic diagram of the OXCT1 promoter with two potential YY1 binding sites. (C) The YY1 binding motif was predicted through bioinformatics analysis. (D) The wild-type reporter plasmid containing the OXCT1 gene and pRL-TK-Renilla was co-transfected with either the YY1 overexpression plasmid or siYY1 into CRC cells, and dual luciferase activity was measured. (E) ChIP-qPCR analysis indicated that YY1 directly bound to the OXCT1 promoter in HCT116 and RKO cells. The results were validated using a t-test. Gene enrichment was assessed relative to input controls using qPCR with primers specific to the promoter regions of OXCT1 (∗*p* < 0.05, ∗∗*p* < 0.01, ∗∗∗*p* < 0.001).

**Figure S3:**
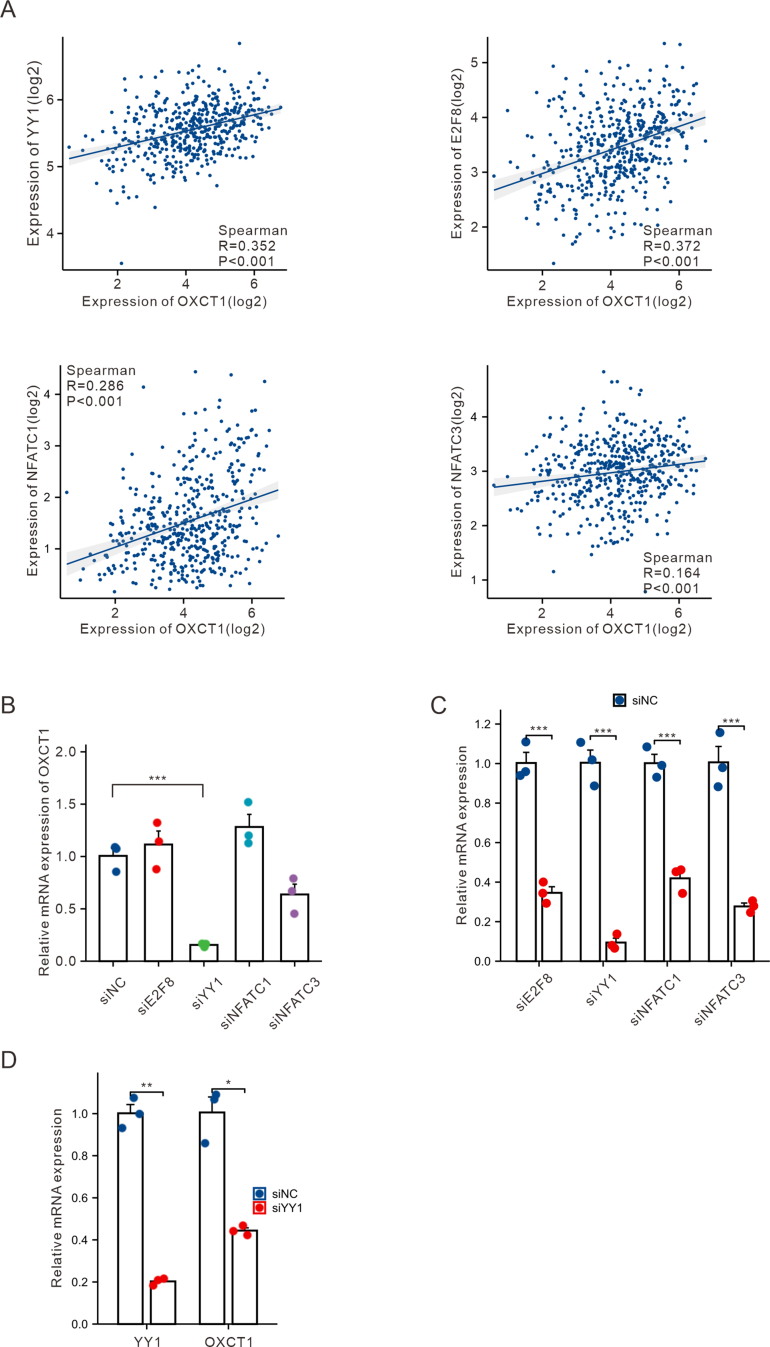
The transcription regulation of OXCT1 by Yin and Yang 1 (YY1). (A) The correlation between transcription factors and OXCT1 was analyzed using TCGA data. (B–C) The impact of knocking down candidate transcription factors on OXCT1 expression at the mRNA level. (D) Knocking down YY1 reduced the expression of OXCT1 at the transcriptional level in RKO cells (∗*p* < 0.05, ∗∗*p* < 0.01, ∗∗∗*p* < 0.001).

To investigate the regulatory mechanisms of OXCT1 expression in colorectal cancer, we performed a comprehensive screening using bioinformatics approaches. Firstly, we used the JASPAR^19^ tool from UCSC^20^ to predict transcription factors that may regulate OXCT1 expression, and found YY1, E2F8, NFATC1, and NFATC3 as potential candidates. We next performed a Spearman correlation analysis^21^ of these genes' expression levels in colon tumor tissues using data from the TCGA database (Fig. S3A). The results showed that all four genes were significantly correlated with OXCT1 mRNA levels. Subsequently, we used siRNA to knock down these four transcription factors, and assessed their impact on OXCT1 expression by qPCR (Fig. S3B-C). Notably, only the knockdown of YY1 significantly decreased the mRNA levels of OXCT1 in both cell lines (Fig. S3D). Furthermore, Western blot confirmed that overexpression of YY1 significantly increased OXCT1 protein levels, while YY1 knockdown reduced OXCT1 protein expression (Fig. 3A). Collectively, these results indicate that YY1 is a key transcription factor of OXCT1 in colorectal cancer.

In addition, we predicted YY1 binding sites within the OXCT1 promoter region using the JASPAR database. And, we found the most probable binding motifs for YY1 were located within the −1191 to −1197, and −1269 to −1275 regions (Fig. 3B–C). To further determine whether YY1 could bind to the promoter region of OXCT1 and regulate its transcriptional activity, we constructed a pGL3-OXCT1 plasmid containing the OXCT1 promoter, and confirmed its activity using a dual-luciferase reporter assay. The results indicated the overexpression and knockdown of YY1 could significantly increase and decrease the luciferase activity, respectively (Fig. 3D). Finally, ChIP-qPCR experiments confirmed these two potential binding sites of YY1 on the OXCT1 promoter (Fig. 3E). In summary, these findings suggested that YY1, as a key upstream regulatory factor, is involved in the transcriptional regulation of OXCT1.

### OXCT1 inhibits colon cancer metastasis by regulating the CDK8-β-catenin axis

**Figure 4:**
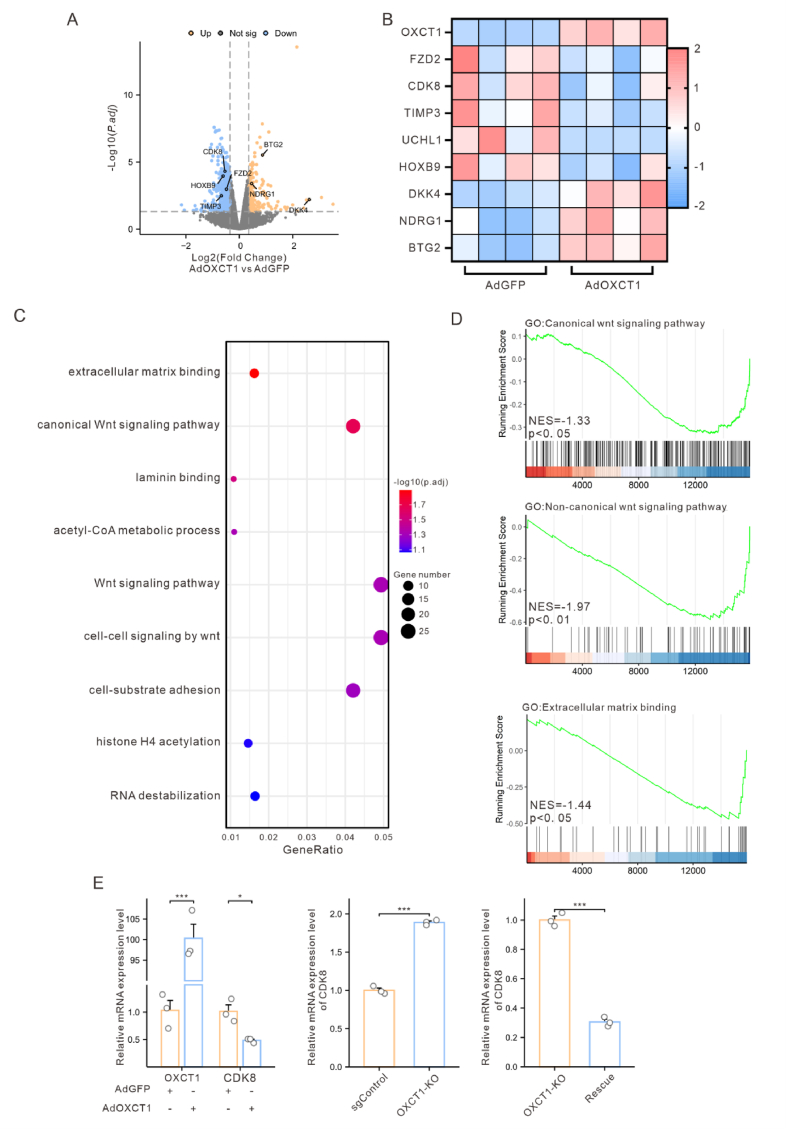
Overexpression of OXCT1 inhibited CDK8 expression in mRNA level. **(A)** Visualization analysis of differential expression in transcriptome sequencing data. **(B)** Heatmap of migration-related genes in DEGs. **(C)** GO enrichment analysis was performed on differentially expressed genes. **(D)** GSEA enrichment analysis was conducted on all genes. **(E)** qPCR validation of the effect of OXCT1 on CDK8 transcription levels (∗*p* < 0.05, ∗∗*p* < 0.01, ∗∗∗*p* < 0.001).

To unravel the mechanisms by which OXCT1 inhibits tumor metastasis, we performed transcriptomic sequencing analysis on HCT116-derived cell lines to find differentially expressed genes (DEGs) between AdGFP and AdOXCT1 groups. By applying thresholds of adjusted *p*-value <0.05 and |logFC| > 0.35, we identified a total of 648 significantly differentially expressed genes (DEGs) (Fig. 4A). A literature review indicated that many of these genes are closely linked to tumor metastasis. Specifically, highly expressed genes in the AdOXCT1 group, such as FZD2,^22^^,^^23^ CDK8,^24^ TIMP3,^24^ and UCHL1,^25^ are known to promote metastasis. In contrast, genes associated with metastasis inhibition, such as DKK4,^26^ NDRG1,^27^ and BTG2,^28^ were significantly upregulated in the AdGFP group (Fig. 4B).

**Figure S4:**
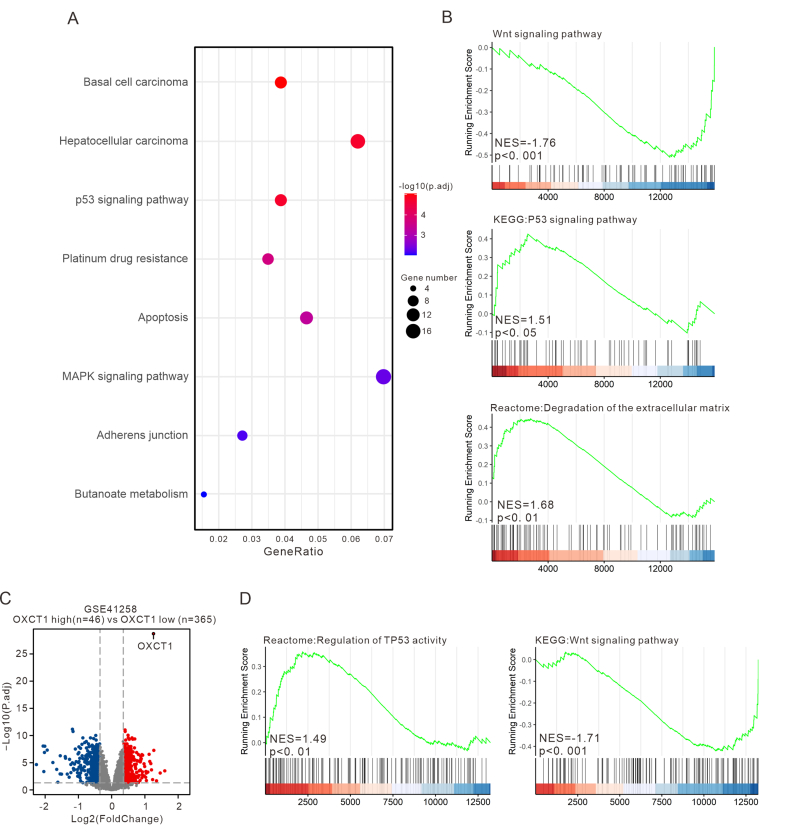
Overexpression of OXCT1 inhibited CDK8 expression in mRNA level. (A) KEGG enrichment analysis was performed on differentially expressed genes. (B) GSEA enrichment analysis was conducted on all genes of RNA-seq. (C) Differential gene expression analysis was performed based on KM analysis grouping for the GSE41258 dataset, and the results were visualized using a volcano plot. (D) GSEA analysis was performed on OXCT1-related differential genes with combined LOGFC for the GSE41258 dataset.

Subsequently, we performed KEGG/GO enrichment analysis and GSEA enrichment analysis on these DEGs. GO enrichment analysis revealed that these genes were primarily enriched in biological processes related to tumor metastasis, such as the Wnt signaling pathway, matrix adhesion-dependent cell spreading, and extracellular matrix binding (Fig. 4C). KEGG enrichment analysis indicated significant enrichment of these genes in signaling pathways such as basal cell carcinoma, hepatocellular carcinoma, p53 signaling pathway, and adherens junctions (Fig. S4A). Additionally, GSEA enrichment analysis further confirmed significant enrichment of the DEGs in the Wnt signaling pathway (Fig. 4D, Fig. S4B).

Further analysis of OXCT1-related DEGs (Fig. S4C) in the GSE41258 dataset similarly showed significant enrichment in the Wnt signaling pathway according to GSEA results (Fig. S4D). Taken together, OXCT1 might inhibit colon cancer metastasis by suppressing the Wnt signaling pathway.

**Figure 5:**
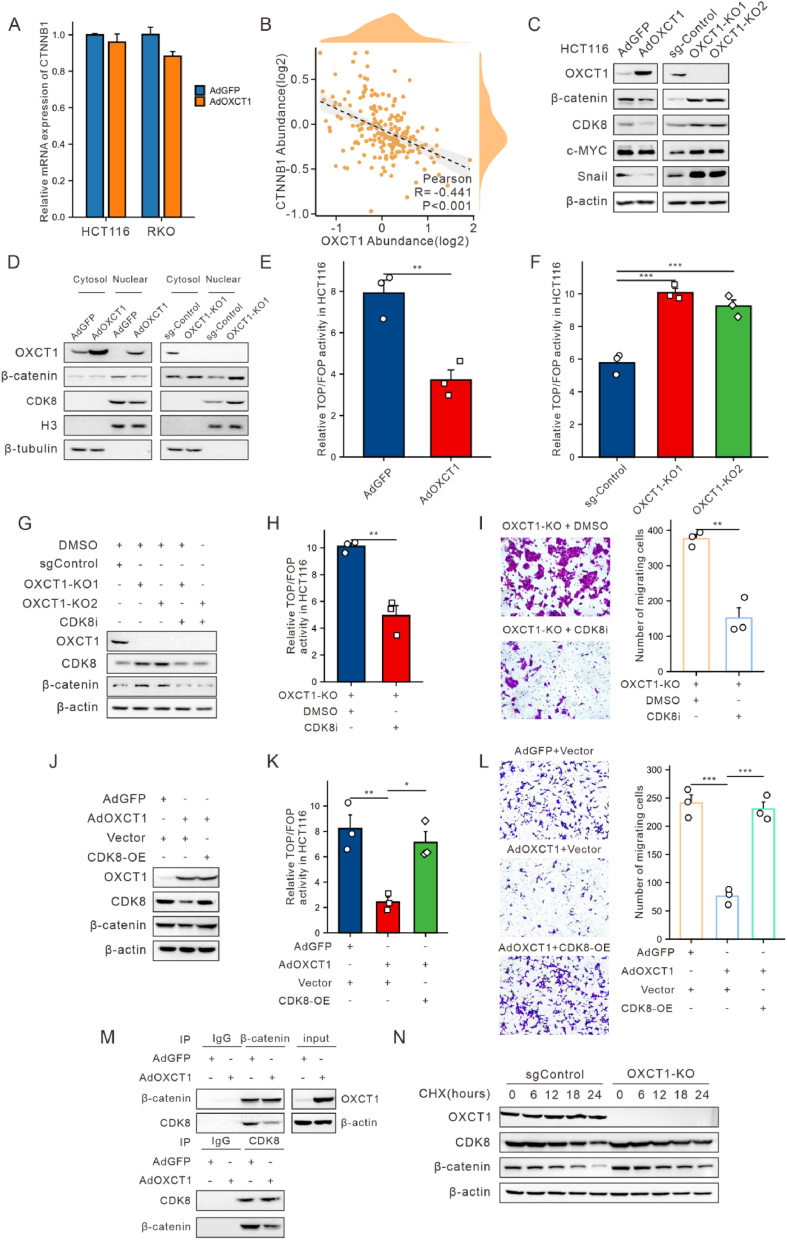
Overexpression of OXCT1 inhibited colon cancer metastasis by regulating the CDK8-β-catenin axis. (A) qPCR study on the effect of OXCT1 on β-catenin transcription levels. (B) CPTAC database prediction of OXCT1's regulation on β-catenin protein levels. (C) Effects of OXCT1 on the WNT pathway in HCT116. (D) Regulation of β-catenin expression in the nucleus by OXCT1. (E–F) Verification of OXCT1's effect on β-catenin transcriptional activity using the TOP/FOPflash dual - luciferase assay in HCT116. (G–H) Inhibition of CDK8 restored the promotion of β-catenin by OXCT1-KO group. (I) The CDK8 inhibitor reversed the enhanced cell migration caused by OXCT1 knockout. (J–K) The overexpression of CDK8 rescued the inhibitory effect of β - catenin in the AdOXCT1 group. (L) The overexpression of CDK8 rescued the inhibitory effect of AdOXCT1 on the migration of HCT116 cells. (M) Investigation of OXCT1's effect on the interaction between CDK8 and β-catenin using IP assay in HCT116. (N) Western blotting analysis of β-catenin in OXCT1-KO treated with 50 μg/mL of CHX. The cells were harvested at the indicated times. The knockout of OXCT1 prolonged the half-life of β-catenin protein (∗*p* < 0.05, ∗∗*p* < 0.01, ∗∗∗*p* < 0.001).

Thus, we validated the expression of CDK8 which was related to the Wnt signaling pathway by qRT-PCR, and found that the transcription level of CDK8 was significantly elevated following OXCT1 knockout, and downregulated after restoration of OXCT1 (Fig. 4E). These results suggested that OXCT1 might regulate the Wnt pathway through CDK8. On the other hand, β-catenin is the key protein in the Wnt signaling pathway.^29^ Thus, we performed qRT-PCR analysis of OXCT1 and β-catenin in each of the AdOXCT1 and AdGFP groups of RKO and HCT116 cell lines, respectively (Fig. 5A). The results indicated that OXCT1 did not significantly affect β-catenin expression at the transcriptional level, but did significantly regulate CDK8 expression.

**Figure S5:**
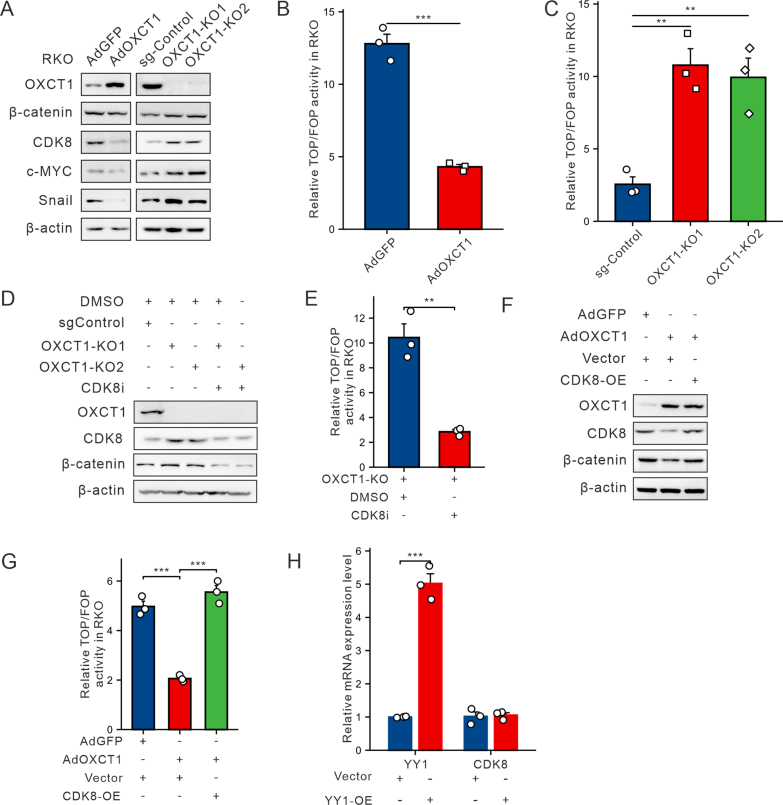
Overexpression of OXCT1 inhibited colon cancer metastasis by regulating the CDK8-β-catenin axis. (A) Effects of OXCT1 on the WNT pathway in RKO. (B–C) Verification of OXCT1's effect on β-catenin transcriptional activity using the TOP/FOPflash dual-luciferase assay in RKO. (D-E) Inhibition of CDK8 restored the promotion of β-catenin by OXCT1-KO group in RKO. (F-G) The overexpression of CDK8 rescued the inhibitory effect of β-catenin in the AdOXCT1 group. (H) The effect of YY1 overexpression on CDK8 mRNA levels (∗*p* < 0.05, ∗∗*p* < 0.01, ∗∗∗*p* < 0.001).

Furthermore, our examination of the CPTAC database^30^ found a significant negative correlation between OXCT1 and β-catenin at the protein levels (Fig. 5B). Further Western blot analysis confirmed that OXCT1 inhibited the protein expression of β-catenin and CDK8 (Fig. 5C, Fig. S5A). These findings suggested that OXCT1 might suppress the protein level of CDK8 and β-catenin of the Wnt signaling pathway.

In addition, we conducted Western blot analysis of cytoplasmic and nuclear proteins to investigate how OXCT1 affects β-catenin in both compartments. The results indicated that OXCT1 significantly inhibited the expression levels of nuclear β-catenin and CDK8 (Fig. 5D). Next, we conducted TOPflash and FOPflash dual-luciferase assays under OXCT1 overexpression and knockout conditions, respectively (Fig. 5E–F, Fig. S5B-C). These assays further confirmed the regulatory effect of OXCT1 on the downstream Wnt signaling pathway.

To verify whether overexpression of OXCT1 inhibits the migratory ability of colon cancer cells via CDK8, we treated the cells with the CDK8 inhibitor (MSC2530818). The results showed that the CDK8 inhibitor reversed the increase of β-catenin levels and TOP/FOP activity caused by OXCT1 knockout (Fig. 5G–H, Fig. S5D-E), and also reduced the enhanced cell migration after the knockout (Fig. 5I). Next, we found that overexpression of CDK8 in the AdOXCT1 group reversed the inhibitory effect of high OXCT1 expression on β-catenin (Fig. 5J–K, Fig. S5F-G). Similarly, CDK8 overexpression reversed the inhibitory effect of OXCT1 on the migration ability of HCT116 cells (Fig. 5L).

Previous studies have found that CDK8 upregulates the expression of β-catenin,^31^ and that the inhibition of β-catenin transcription by E2F1 is counteracted by CDK8.^32^ To further investigate the relationship between CDK8 and β-catenin, we conducted Co-IP experiments using AdGFP and AdOXCT1, respectively. Specifically, when IP was performed for β-catenin (with normalized β-catenin), a significant decrease in CDK8 levels was observed. Conversely, when IP was performed for CDK8 (with normalized CDK8), we observed a decrease in β-catenin levels. These results showed that overexpression of OXCT1 downregulated the levels of CDK8 and β-catenin, and reduced their interaction (Fig. 5M). Additionally, the half-life of β-catenin was significantly prolonged in OXCT1-KO HCT116 cells after CHX treatment (Fig. 5N). These findings suggested that OXCT1 influenced the stability of CDK8. Furthermore, we found that YY1, as a transcription factor, does not regulate the mRNA level of CDK8, a downstream target of OXCT1 (Fig. S5H). This suggested that YY1 may not be involved in the regulatory mechanisms of OXCT1 on its downstream pathways, which likely involve a complex regulatory network.

### OXCT1 suppresses CDK8 expression by reducing H3 protein acetylation

OXCT1 is a key enzyme in ketone body metabolism, and its enzymatic activity depends on the S226 amino acid residue. It was found that mutating this amino acid residue to S226N abolished OXCT1's ability to inhibit the migration of colon cancer cells.^33^ Drawing from previous research by Zhu et al.,^34^ which indicated that OXCT1 enhances H3 protein acetylation by regulating ketone body metabolism, we conducted a series of experiments.

**Figure 6:**
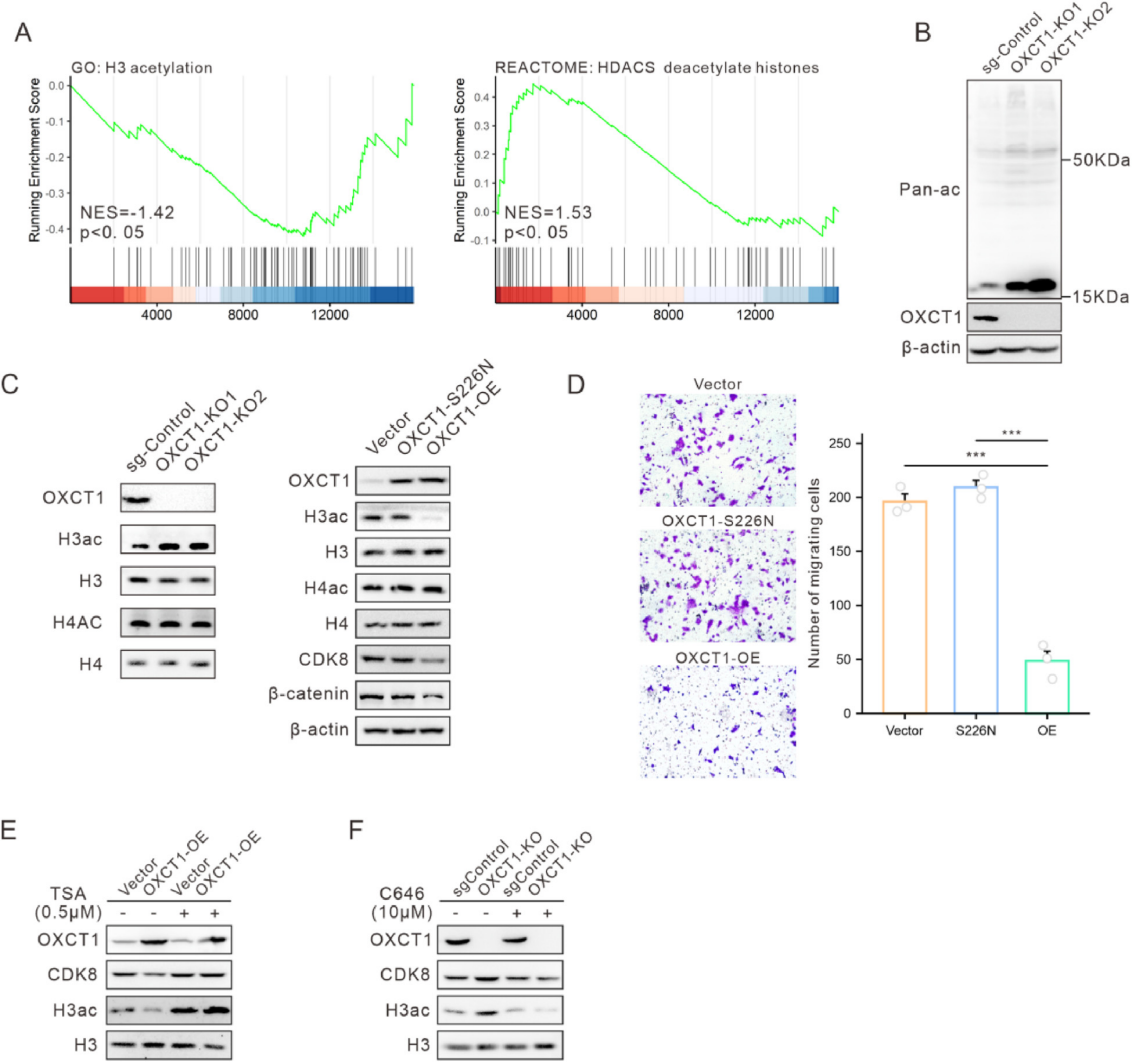
OXCT1 modulated CDK8 expression by regulating H3 protein acetylation. **(A)** GSEA analysis of RNA-seq results showed that OXCT1 overexpression inhibited H3 acetylation and the degradation of HDACs. **(B)** OXCT1 knockout promoted an increase in the acetylation of small molecular weight proteins, as demonstrated by Western blot experiments. **(C)** Western blotting was used to detect the levels of histone H3 acetylation and histone H4 acetylation in the OXCT1 overexpression, mutation, and knockout groups of HCT116 cells. **(D)** The inhibitory effect on HCT116 cell migration was lost after the enzymatic inactivation mutation of OXCT1. **(E–F)** This study investigated the effects of Trichostatin A (TSA) and C646 on H3ac levels and CDK8 expression in HCT116 cells. Two experimental groups were set up: an overexpression group with or without 0.5 mM TSA treatment, and a knockout group with or without 10 mM C646 treatment (∗*p* < 0.05, ∗∗*p* < 0.01, ∗∗∗*p* < 0.001).

First, we performed GSEA analysis on RNA-seq data and found that the results were significantly associated with H3 acetylation (Fig. 6A). We examined the global histone acetylation levels following OXCT1 knockout, and found a significant increase in the expression of low-molecular-weight proteins (Fig. 6B). Further experiments revealed that H3 protein acetylation levels significantly increased after OXCT1 knockout, whereas H4 protein acetylation levels remained largely unchanged. Overexpression of OXCT1 inhibited the acetylation of H3 protein and the CDK8-β-catenin axis, whereas overexpression of the OXCT1-S226N mutant did not affect the inhibition of H3 protein acetylation and the CDK8-β-catenin axis (Fig. 6C). Additionally, we found that the OXCT1-S226N mutant did not influence the migratory ability of HCT116 cells (Fig. 6D). These findings suggest that OXCT1 inhibits H3 acetylation through its enzymatic activity and, consequently, suppresses the migration ability of colon cancer cells.

**Figure 7:**
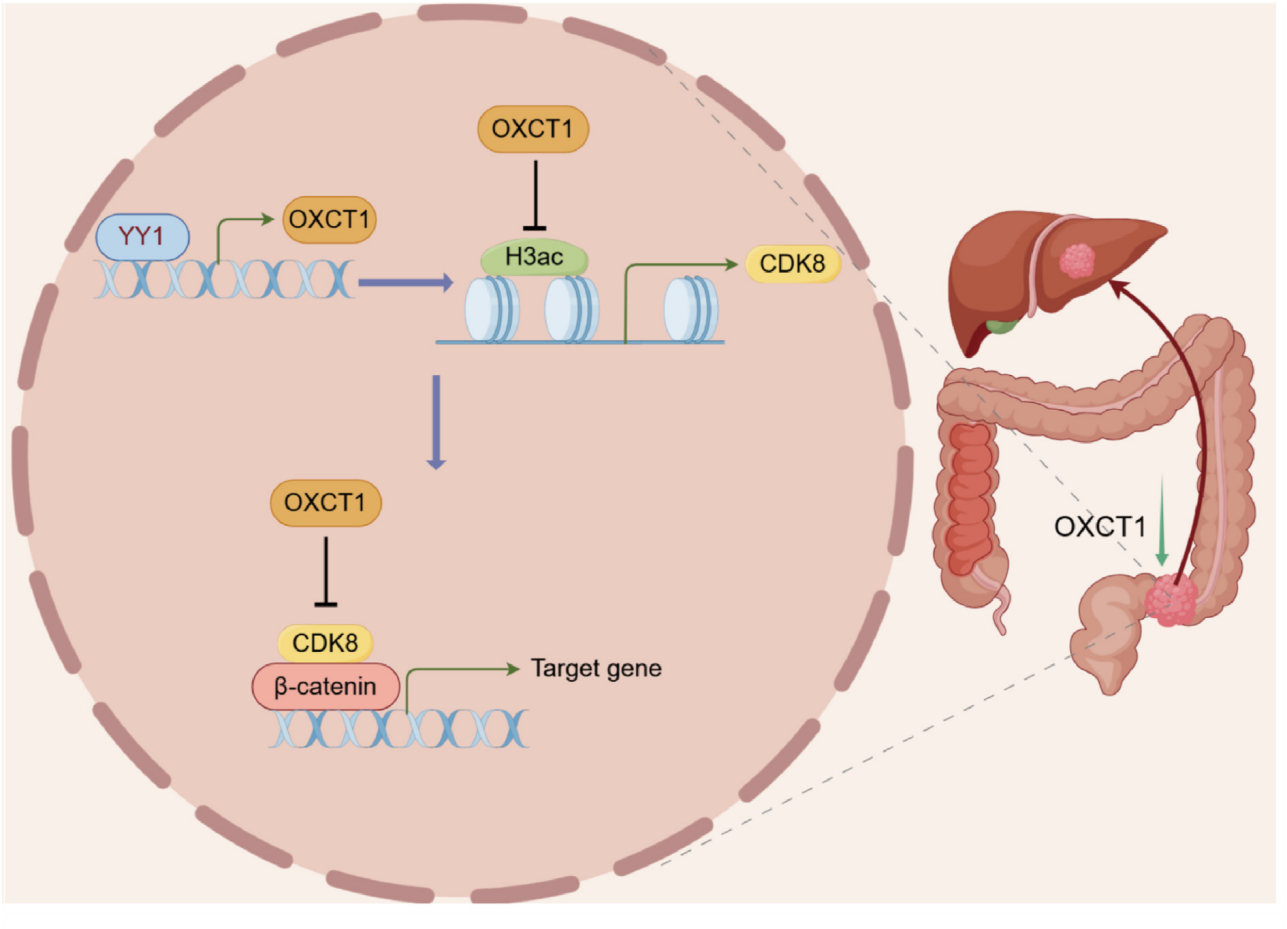
In colorectal cancer, OXCT1 is a suppressor of tumor progression, and low expression of OXCT1 is associated with poor prognosis. The transcriptional level of OXCT1 is promoted by YY1, and OXCT1 inhibits the expression of CDK8 and β-catenin by suppressing H3 acetylation, thereby further inhibiting liver metastasis of colorectal cancer.

To investigate whether OXCT1 regulates CDK8 expression depending on H3 acetylation, we treated cells with the histone deacetylase (HDAC) inhibitor Trichostatin A (TSA), and the p300 acetyltransferase inhibitor C646 to modulate H3 acetylation levels. Results showed that TSA treatment blocked the effect of OXCT1 overexpression on the reduction of H3 acetylation levels, and downregulation of CDK8 (Fig. 6E), while C646 treatment inhibited the effect of OXCT1 knockout on the increase of H3 acetylation levels, and upregulation of CDK8 in HCT116 cells (Fig. 6F). In conclusion, OXCT1 suppresses H3 acetylation through its enzymatic activity, which in turn downregulates CDK8 expression.

**Figure S6:**
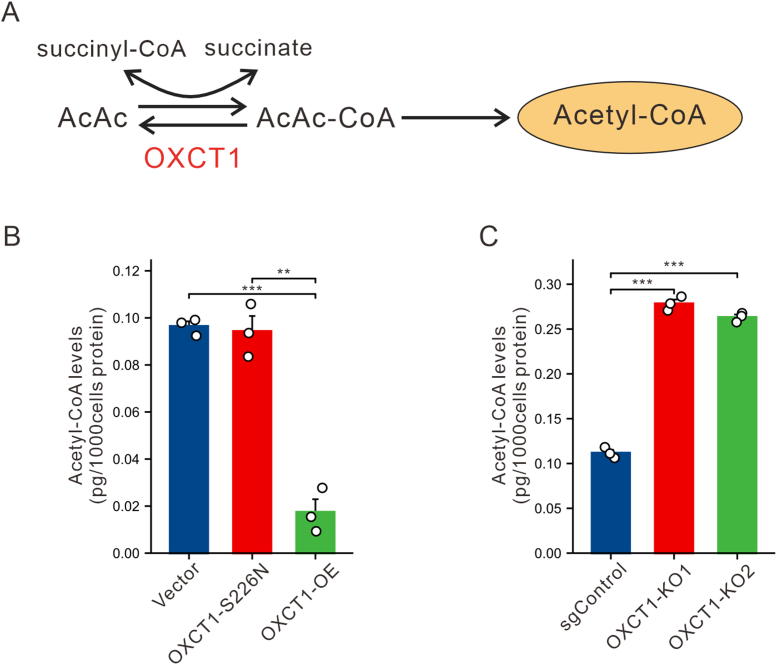
OXCT1 regulates H3 histone acetylation by modulating the concentration of acetyl-CoA. (A) Diagram of OXCT1 in Ketones metabolism. (B–C) The levels of acetyl-CoA in HCT116 cells with OXCT1 overexpression (B) and knockout (C) (∗*p* < 0.05, ∗∗*p* < 0.01, ∗∗∗*p* < 0.001).

As a metabolic enzyme, OXCT1 mediates the reversible transfer of coenzyme A (CoA) between acetoacetate (AcAc) and succinyl-CoA. Typically, OXCT1 is thought to regulate the production of acetyl-CoA from AcAc (Fig. S6A). Interestingly, we observed that acetyl-CoA levels decreased following OXCT1 overexpression, a phenomenon dependent on OXCT1's enzymatic activity, as evidenced by the S226N mutation (Fig. S6B-C). These results suggested that, in the context of colon cancer, OXCT1 might preferentially drive the conversion of acetoacetyl-CoA to AcAc.

## Discussion

Colorectal cancer (CRC) is one of the most common malignant tumors of the digestive system worldwide. Metastasis is the primary cause of poor prognosis in patients. Extensive research has focused on the mechanisms of migration, however, the specific molecular mechanisms of CRC metastasis are still not fully understood. In this study, we used bioinformatics methods to analyze DEGs associated with colorectal cancer in the GSE41258 dataset, And we performed univariate Cox regression analysis, LASSO regression analysis, along with survival analysis to further screen for prognosis-related genes. The results indicated that OXCT1 was the only gene that was downregulated in these datasets. We conducted overexpression and knockout experiments of OXCT1 in colorectal cancer cells. Results showed that OXCT1 overexpression significantly inhibited CRC metastasis, suggesting that OXCT1 might serve as a protective factor against colorectal cancer. This finding contrasted sharply with previous studies.^16^^,^^17^^,^^35^

OXCT1 (3-ketoacid CoA transferase 1) is a protein composed of 521 amino acids located in the mitochondrial matrix.^36^ It is widely expressed in the heart, brain, and kidneys, but is nearly undetectable in liver tissue.^37^ Previous studies have shown that OXCT1 was a key factor in promoting tumor progression and drug resistance in liver, lung, and pancreatic cancers, and was closely related to energy metabolism, proliferation, and survival of cancer cells. Despite being reported as a critical factor in ketogenic diet therapy for colorectal cancer, its impact on CRC metastasis remains unclear.^38^ We explored OXCT1 and other key genes involved in CRC metastasis from different perspectives, which might help reveal potential targets for CRC metastasis, and provide guidance for clinical medication and personalized therapy.

Previous studies have found that the abnormal Wnt signaling pathway, resulting from the interaction between the APC gene and β-catenin protein,^39^^,^^40^ was crucial in both the initial stages of CRC metastasis, and the later stages of migration and invasion.^41^ Inhibiting the Wnt signaling pathway decreases tumor proliferation, migration, invasion, and angiogenesis, highlighting its potential as a target for slowing CRC progression. In our study, by comparing the transcriptome sequencing of OXCT1 overexpression and control groups, we found that OXCT1 regulates the expression of CDK8 at the mRNA level. CDK8 is a member of the cyclin-dependent kinase complex, responsible for coupling transcriptional regulators with the basal transcription machinery.^42^ The CDK8 gene is frequently amplified in CRC, and its kinase activity is crucial for regulating β-catenin-dependent transcription.^43^ We found that OXCT1 inhibition affects β-catenin protein expression by regulating CDK8 transcription, which further influences the activation of the Wnt signaling pathway. Additionally, we discovered that OXCT1 regulates the interaction between β-catenin and CDK8. Notably, the regulatory effect of OXCT1 on tumor cell migration could be reversed by a CDK8 inhibitor. This finding provided new insights into the role of OXCT1 in CRC metastasis, and suggested that CDK8 might be a potential therapeutic target.

As a metabolic enzyme, OXCT1 can catalyze the transfer of CoA from succinyl-CoA, forming a reversible reaction process with acetoacetyl-CoA, thereby regulating the production of metabolites such as acetyl-CoA and succinyl-CoA.^44^ This led us to consider how OXCT1 regulates CDK8 transcription. Previous studies have indicated that metabolite changes induced by OXCT1 can affect histone H3 acetylation in the nucleus.^34^ In our study, we found that in CRC, OXCT1 regulated CDK8 expression by inhibiting the histone acetylation in colorectal cancer cells. This regulation depends on the enzymatic activity of OXCT1. Therefore, we believe that OXCT1 may inhibit the activation of the Wnt signaling pathway by regulating the balance of intracellular metabolites, and suppressing histone H3 acetylation. Next, we found that acetyl-CoA levels decreased upon OXCT1 overexpression. This effect was dependent on OXCT1's enzymatic activity, as demonstrated by the S226N mutation. Previously research has indicated that acetyl-CoA metabolism regulated histone acetylation modifications in cancer, and the overall intracellular levels of acetyl-CoA were positively correlated with histone modification levels. An increase in acetyl-CoA concentration enhanced the activity of lysine acetyltransferases (KATs), thereby promoting histone acetylation.^45^ In summary, we proposed that OXCT1 regulated cellular migration ability by modulating intracellular acetyl-CoA levels, which in turn affect histone acetylation and subsequently regulate CDK8 transcription.

Furthermore, we found that OXCT1 could regulate the interaction between CDK8 and β-catenin. These findings suggest that OXCT1's regulation of CRC metastasis involves a complex network among OXCT1, CDK8, and Wnt signaling pathway as illustrated in the mechanistic figure. Based on our results, CDK8 might be a therapeutic target of CRLM in case of low OXCT1 expression. Additionally, overexpression of OXCT1 might be utilized in the cell therapy under conditions of low OXCT1 expression.

This study has several limitations. First, although YY1 regulates OXCT1, it did not modulate the Wnt signaling pathway downstream of OXCT1, which did not fully explain the downregulation of OXCT1 in CRLM. Additionally, the regulation of OXCT1 by YY1 might involve a complex regulatory network. Second, the mechanistic insights into how OXCT1 regulates histone acetylation remain incomplete. In future studies, we will investigate OXCT1-mediated metabolomic changes to provide a more comprehensive understanding of the molecular mechanisms by which OXCT1 regulates CRLM.

## CRediT authorship contribution statement

**Chenhao Li:** Writing – original draft, Software, Methodology, Investigation, Data curation. **Deao Gong:** Methodology, Data curation. **Xiaoqun Shan:** Software, Methodology. **Kang Wu:** Validation. **Jiayao Yang:** Formal analysis. **Rong Zhang:** Methodology. **Ye Huang:** Formal analysis. **Kai Wang:** Writing – review & editing, Project administration. **Ni Tang:** Supervision, Project administration. **Yuxi Zhu:** Writing – review & editing, Writing – original draft, Investigation.

## Ethical approval

This study was designed with human participants in accordance with the Declaration of Helsinki and received approval from the Medical Research Ethics Committee of the First Affiliated Hospital of Chongqing Medical University (Approval No. 2024-031-01).

The animal experiments involved in this project were reviewed by the Institutional Animal Care and Use Committee of Chongqing Medical University (IACUC-CQMU) and comply with the principles of animal protection, welfare, and ethics (Approval No. IACUC-CQMU-2024-0654).

## Data available statement

Both the data and datasets used in this study are available in the online database TCGA and GEO. GEO Accession Numbers: GSE41258, GSE68468, GSE35834.

## Funding

This study was funded by Special Key Project of Chongqing Technology Innovation and Application Development, 10.13039/501100002865Chongqing Science and Technology Bureau, China (CSTB2023TIAD-KPX0006).

## Conflict of interests

Ni Tang is one of the Associate Editors of *Genes & Diseases*, but he/she has no involvement in the peer-review of this article and has no access to information regarding its peer-review. No other potential conflict of interests should be declared.
