# Supplementary material for: Low expression of OXCT1 promote colorectal cancer liver metastasis by upregulating CDK8 and β-catenin via H3 acetylation
Source: Genes Dis. 2025 Apr 9;13(2):101625. doi: 10.1016/j.gendis.2025.101625 (PMC12765264; doi:10.1016/j.gendis.2025.101625)
Supplement: Multimedia component 1 [file mmc1.docx]

Table S1: Details of the GEO and TCGA databases used in this study

| **GEO ID** | **Platform** | **Numbers of patients** | **Clinical data** |
| --- | --- | --- | --- |
| GSE41258 | GPL96 | Primary tumor: 186  (M0:127, M1:58)  Liver mets: 47 | Overall survival(OS)  in primary tumor |
| GSE68468 | GPL96 | Primary tumor: 195  Liver mets: 47 |  |
| GSE35834 | GPL15236 | Primary tumor: 31  Liver mets: 27 |  |
| TCGA-COAD | Illumina RNAseq | Primary tumor: 474  (M0:353, M1:67) | Overall survival(OS)  in primary tumor |
